# Psychometric Properties of the Spanish Versions of EQ-5D-Y-3L and EQ-5D-Y-5L in Children with Cancer: A Comparative Study

**DOI:** 10.3390/ijerph191811420

**Published:** 2022-09-10

**Authors:** Miguel A. Perez-Sousa, Pedro R. Olivares, Narcis Gusi

**Affiliations:** 1 Department of Specific Didactics, Faculty of Education, University of Córdoba, 14071 Cordoba, Spain; 2 Epidemiology of Physical Activity and Fitness across Lifespan Research Group, University of Seville, 41004 Seville, Spain; 3 Faculty of Education, Psychology and Sport Sciences, University of Huelva, Avenida de las Fuerzas Armadas s/n, 21007 Huelva, Spain; 4 Instituto de Actividad Fisica y Salud, Universidad Autonoma de Chile, 3460000 Talca, Chile; 5 Faculty of Sport Sciences, University of Extremadura, Avenida de la Universidad s/n, 10003 Cáceres, Spain

**Keywords:** cancer, children, EQ-5D-Y-5L, health-related quality of life, psychometric properties

## Abstract

(1) Background: The recent published version with five levels of response of EQ-5D-Y needs to be studied in children with chronic illness. For this, the aim of the present study was to assess and compare the psychometric properties of EQ-5D-Y-3L and EQ-5D-Y-5L in terms of feasibility, ceiling effect, redistribution properties, informativity and inconsistence responses in children with cancer. (2) Methods: A core set of self-report tools, including the Spanish version of EQ-5D-Y-3L and EQ-5D-Y-5L, were administered to children drawn from the population with cancer. EQ-5D-Y-3L and EQ-5D-Y-5L were evaluated in terms of feasibility, ceiling effects, redistribution properties and differences in absolute and relative informativity. (3) Results: A total of 73 children (9.7 ± 2.3 years old) from the population with cancer participated in the study. No missing data in the new EQ-5D-Y-5L were visualized, so the feasibility was acceptable. EQ-5D-Y-5L showed a low ceiling effect in all dimensions with relative changes from EQ-5D-Y-3L to EQ-5D-Y-5L of between 15.3% and 42.4% for the dimensions and 44.6% for the overall system. Compared to EQ-5D-Y-3L, EQ-5D-Y-5L provided a better distribution of the severity of the problem in the five levels of response. The absolute informativity (Shannon’s index) did not show statistically significant differences between EQ-5D-Y-3L and EQ-5D-Y-5L in all dimensions and the overall system. (4) Conclusions: EQ-5D-Y-5L is feasible, presenting a low ceiling effect and high discriminative power.

## 1. Introduction

The importance of understanding the impact of disease and treatment on children has been increasingly requested in clinical practice. To do this, it is necessary to know the perception that the child has about their state of health and how the disease and the treatment affect their Health-related Quality of Life (HRQoL) [1]. HRQoL is a complex, multidimensional concept, including social, emotional and physical functioning or well-being, and is influenced by a person’s objective assessments of their health status and function as well as subjective perceptions of their personal health [2,3]. The increase in knowing a child’s perception in paediatric clinical trials has led to a greater variety and use of questionnaires for the assessment of HRQoL. There now exist various validated generic HRQoL questionnaires for use with children, such as the Paediatric Quality of Life Inventory (PedsQL) [4], KIDSCREEN [5] and EQ-5D-Y-3L. However, these instruments face the challenge of being able to assess the HRQoL of the general population and specific illness subgroups considering the continuous physical, emotional, social and cognitive development during childhood and adolescence.

EQ-5D-Y-3L is a generic child-friendly self-completed and widely used instrument to measure and evaluate HRQoL in children and adolescents aged from 8 to 15 years [6]. EQ-5D-Y-3L has a descriptive system which comprises five dimensions: mobility (“walking about”), self-care (“looking after myself”), usual activities (“doing usual activities”), pain or discomfort (“having pain or discomfort”) and anxiety or depression (“feeling worried, sad or unhappy”). Each dimension presents three levels of problems using the wording “no problems” (level 1), “some problems” (level 2), and “a lot of problems” (level 3) [7]. EQ-5D-Y-3L has demonstrated its feasibility in children and adolescents with different health conditions [8,9]. However, EQ-5D-Y-3L presents some issues, such as a higher ceiling effect [6] and an inability to detect changes in health status [10,11]. Euroqol Research Group, taking into account these limitations, recently developed a new version increasing the number of response levels to five, i.e., the ‘EQ-5D-Y-5L’ instrument [12]. To date, a few studies have studied the psychometric properties of this instrument. Pérez-Sousa et al. [13] showed that EQ-5D-Y-5L, in the general population, was feasible, consistent and reliable. However, they found a scarce difference between the three-level and five-level versions in terms of their informativity and ceiling effect. Fitriana et al. [14] indicated that EQ-5D-Y-5L had small improvements in its psychometric performance compared to EQ-5D-Y-3L in paediatric patients.

Therefore, the EQ-5D-Y-5L version needs to be explored in children with chronic illnesses. In this sense, cancer is a disease with a severe physical and psychosocial burden and, in recent years, with the aim of improving care in these patients, emphasis has been placed on assessing the HRQoL [15].

The study of the psychometric properties of the new EQ-5D-Y-5L questionnaire and its comparison with EQ-5D-Y-3L is indispensable for the validity of future studies in child patients with chronic illness.

Therefore, we conducted this study to examine the psychometric properties of EQ-5D-Y-5L and compare the performance with EQ-5D-Y-3L in terms of its feasibility, ceiling effect, redistribution properties, inconsistency and informativity.

## 2. Materials and Methods

### 2.1. Study Design

This was a cross-sectional study.

### 2.2. Sample and Setting

The study was conducted between February 2017 and December 2020. Participants were recruited from the Regional Association of Parents of Children with Cancer (ANDEX) located in Sevilla, Spain. All newly diagnosed children cared for in the participating association were assessed for eligibility. Inclusion criteria were patients 6–18 years old with a diagnosis of cancer. Participants had to be able to understand spoken Spanish. No exclusion criteria were present. We included children aged 6–14 years. There were 3 diagnostic groups involved: haematological cancer (leukemia and lymphomas), solid tumours (malignant bone tumours, soft tissue and other extra osseous sarcomas, neuroblastoma and other peripheral nervous cell tumours or renal tumours) and brain tumours. 

### 2.3. Ethics

Before data collection, the parents were informed of the study’s methodology and objectives through an official letter written by the researchers that included an informed consent form. To be included in the study, written consent from parents and verbal assent from children were obtained. Likewise, they had the right to withdraw consent to participate in the study at any time without explanation. The study was approved by the Bioethics Committee of Universidad de Extremadura and was conducted following the International Ethical Guidelines for Biomedical Research Involving Human Subjects, established in Geneva.

### 2.4. Instruments

All participants completed a paper-based survey, which included sociodemographic and health questions and several instruments for the measurement of HRQOL in children and adolescents, including the Spanish version of EQ-5D-Y (with 5L and 3L levels of severity) and the EQ visual analog scale (VAS) for young people.

#### 2.4.1. Sociodemographic and Health Measures

Information about age, sex, school grade, type of tumours, age from diagnosis and treatment was collected at the time of data collection.

#### 2.4.2. EQ-5D-Y-3L and EQ-5D-Y-5L

EQ-5D-Y-3L is a generic instrument with 5 dimensions referring to “mobility”, “looking after myself”, “doing usual activities”, “having pain or discomfort” and “feeling worried, sad, or unhappy”. This standard version has 3 severity levels: no problems, some problems, and a lot of problems [6]. EQ-5D-Y-5L has the same dimensions but 5 levels of response (severity): no problems, a little bit of problems, some problems, a lot of problems, and cannot/extreme problems [12]. The Spanish version of this questionnaire has been recently validated [13].

#### 2.4.3. EQ-VAS

Both EQ-5D-Y-3L and EQ-5D-Y-5L include a Visual Analogue Scale (VAS) where the interviewee can report on their health status “today,” in a range of scores from 0 to 100, where 0 indicates the worst health status and 100 represents the best health status. 

### 2.5. Data Collection

The set of questionnaires was administered after cancer diagnosis. The interview was conducted face-to-face by a technician with experience in similar studies, in which the patients had to respond to the items after an explanation by the technician of the procedure and the steps to be followed. The order of the questionnaires in the set was aleatory, where half of children filled out the -3L version first and another half the -5L version. For confidentiality and to facilitate data analysis, each respondent was assigned a code. A phone number and email address were provided to respondents to address any concerns that may arise.

### 2.6. Analysis

All data are presented as mean and standard deviation for continuous variables and frequencies and percentages for categorical variables. Feasibility was examined by calculating the number of missing values for the 3L and 5L versions. The ceiling effect of EQ-5D was defined as the proportion of “no problem” responses in each dimension and in all dimensions. A reduction in the ceiling effect suggested an enhanced classification efficiency. We examined the absolute reduction, calculated as the difference between the proportions of the ceiling effect in both systems. The relative reduction was calculated with the following formula [16]: ceiling 3L—ceiling 5L/ceiling 3L × 100.

Redistribution properties and (in)consistency of responses were evaluated using the method applied in previous studies [13,16] which were described as the proportions of the 3L-5L response pairs within each 3L response level (i.e., 3L-1, 3L-2 and 3L-3). An inconsistent response pair was defined as a 3L response that was at least 2 levels away from the 5L response (e.g., a child chose level 1 [no problem] in the 3L version but responded 3 [moderate problems] in the 5L version); the other pairs were regarded as consistent. The size of inconsistency was calculated as |3L − 5L| − 1, after recoding the EQ-5D-3L responses on the EQ-5D-5L scale (1 = 1; 2 = 3, 3 = 5). We calculated the proportion of each consistent pair in each 3L response level and the percentage of inconsistent pairs in each dimension in addition to their corresponding mean and median VAS values. Our hypothesis was to find the decrease in the mean and median VAS values when moving to lower-status pairs in each dimension [17]; the linear trend was examined through the nonparametric Jonckheere trend test.

The informativity power determined the degree of uniform distribution of responses in each dimension. The more evenly the answers were distributed, the more useful the questionnaire was. We used the Shannon index (*H*′) and the Shannon evenness index (J′) of informativity to compare the discriminatory power of the 3L and 5L versions according to the dimensions and overall system. Shannon’s methodology and indices, originally from the information theory, were applied to the classification and health state mainly for EQ-5D [16,18]. The Shannon index is defined as follows:

(1) $$H^{\prime }=-\overset{L}{\underset{i=1}{\sum }}p_{i}log_{2}p_{i}$$

where *H*′ represents the absolute amount of informativity captured, *L* is the number of possible levels, and *p_i_* is the level of responses in the *i*th level. The higher the *H*′ is, the more the information is captured by the system. Informativity is dependent on the number of response options and the distribution of the observations across levels. In the case of an even (or rectangular) distribution, i.e., if all levels are equally filled, the optimal amount of information is captured, and the Shannon index has reached its upper limit (*H*′max), which is presented by the following formula: *H*′max = log_2_C. For example, *H*′max for the 5L version was log_2_5 or 2.32 and for the 3L version was log_2_3 or 1.58. If the number of levels is increased, *H*′max increases accordingly. The Shannon *t* test [19] was computed to test statistically significant differences (*p* < 0.05) between versions. J′ is constrained between 0 and 1. The less evenness in the responses, the lower J′ is, and vice-versa. J′ is calculated as J′ = *H*′/*H*′max, indicating the usage of the system (*H*′), given its inherent capacity (*H*′max). The 95% confidence intervals for *H*′ were estimated using a non-parametric bootstrap method. Our hypothesis was that the 5L version had more discriminatory power (larger *H*′values) than the 3L version. On the other hand, the Shannon Evenness index J′ reflected that populations needed a larger spread to cover five levels than for three. Therefore, we expected the *H*′ to increase (higher absolute levels of information) and J′ to stay equal or marginally decrease in the 5L version.

## 3. Results

The characteristics of the study sample are presented in Table 1. A total of 73 children (9.7 ± 2.3 years old) completed the survey, of whom 47.9% were males and 52.1% females. The most frequent cancer was haematological (50.7%), followed by solid tumours (37.0%) and brain tumours (12.3%), of which 41.1% had been a year since diagnosis, 32.9% two years and 26% three or more years since diagnosis.

### 3.1. Feasibility and Ceiling Effect

Table 2 shows the frequencies and percentages of the reported problems by the sample using EQ-5D-Y-5L and EQ-5D-Y-3L. The first level of severity (no problems) collated the most responses in all dimensions in EQ-5D-Y-5L rather than EQ-5D-Y-3L. On the second level of severity, it was also observed that this concentrated a high percentage of responses. The rest of the responses were distributed between levels three and four, with hardly children scoring at the level of greatest severity of the problem. There were no missing answers for either EQ-5D-Y-3L or EQ-5D-Y-5L, indicating excellent feasibility for both instruments. Table 3 shows the proportions of “no problems” responses for the EQ-5D-Y-3L and EQ-5D-Y-5L systems and the ceiling effect change. A reduction in the ceiling effect was observed in EQ-5D-Y-5L with respect to EQ-5D-Y-3L in all dimensions and overall. The dimension that reduced the ceiling effect the least was ‘mobility’, with a relative 15.3% and the greatest reduction was in ‘doing usual activities’ with a relative 42.4%. On the other hand, the overall category (11111) decreased by a relative 44.6%.

### 3.2. Redistribution Properties

Table 4 shows the score redistribution from EQ-5D-Y-3L to EQ-5D-Y-5L. Most of the patients who reported a score of one on EQ-5D-Y-3L also reported a score of one on the EQ-5D-Y-5L version. However, there was a significant number of patients who reported a score of one on EQ-5D-Y-3L, reported a score of two (a little bit of problems) on EQ-5D-Y-5L, with percentages of 23.3% in the ‘mobility’, 32.9% in ‘usual activities’ and 27.4% in ‘pain or discomfort’ dimensions. In addition, it was found that a significant proportion of patients who reported a score of two on EQ-5D-Y-3L, reported a score of three on EQ-5D-Y-5L, particularly in the dimensions ‘mobility’ and ‘pain or discomfort’, both with 15.1%. The inconsistencies were scarce in all dimensions, ranging from 1.4% in ‘mobility’, ‘usual activities’ and ‘pain or discomfort’ to 4.1% in ’self-care’ and ‘anxiety/depression’.

### 3.3. Informativity 

Table 5 shows the absolute and relative informativity results of EQ-5D-Y-3L and EQ-5D-Y-5L. The Shannon Index showed a scarce difference in informativity between EQ-5D-Y-3L and EQ-5D-Y-5L. In fact, according to the Shannon *t* test, no statistically significant differences (*p* ≥ 0.05) were obtained between the versions. Likewise, there were hardly any differences in the relative information.

## 4. Discussion

This cross-sectional study compared the psychometric properties of the EQ-5D-Y-3L and EQ-5D-Y-5L instruments in terms of the feasibility, ceiling effect, redistribution properties, inconsistency and informativity in a sample of children with cancer.

The feasibility in both the EQ-5D-Y-3L and EQ-5D-Y-5L versions was excellent since there were not any missing responses. In this way, it seems that there were no comprehensibility problems between both versions in children with cancer. This good performance in acceptability has also been indicated in studies carried out in the general population [13] and in children with different pathologies [14]. 

Ceiling effects were reported in both of the EQ-5D-Y versions, generally presenting a lower size than in other studies on the general population [13,20] but a similar ceiling effect as another study on chronic illness children [14]. In our study, the ceiling effect decreased in EQ-5D-Y-5L with regard to EQ-5D-Y-3L by about 15.3% and 42.4% in the descriptive system and 44.6% in the overall score. This drop was much larger than that reported in other studies [13,20]. It appears that EQ-5D-Y is not able to discriminate among levels of severity in the general population, especially when detecting mild problems. However, in our study, EQ-5D-Y-5L detected the severity of mild problems with major accuracy, i.e., on the levels ‘A little bit of a problem’ and ‘Some problems’. This could be due to the better scaling of the five-response level version.

The redistribution properties from ‘no problems’ in EQ-5D-Y-3L to ‘no problems’ in EQ-5D-Y-5L was larger. We found a proportion ranging from 58.6% in ‘usual activities’ to 81.2% in ‘self-care’. This redistribution was greater compared to similar studies. For example, Fitriana et al. [14] showed a proportion of 83–97% and Pei et al. [20] found a proportion in the redistribution from EQ-5D-Y-3L to EQ-5D-Y-5L of level one responses between 82–98%. This could mean that, in our study, the version with five response levels better detected the health status with small or moderate problems, since the subjects that selected ‘no problems’ in EQ-5D-Y-3L subsequently reported ‘no problems’ and ‘a little bit of problems’ in EQ-5D-Y-5L. The fact of being able to select the health status between ‘no problems’ and ‘a little bit of problems’ was one of the main reasons why the ceiling effect was reduced compared to the EQ-5D-Y-3L version. The redistribution in the middle of scale, i.e., from level two to level two-three-four seemed to be similar to previous studies [14,20]. Inconsistencies were scarce, ranging from 1.4% to 4.1%. These results are in accordance with previous studies in children [20] and adults [17,21] but lower than a study in children with chronic illness [14].

There were three potential weaknesses of this study that need attention. The first is related with the sample size. Compared with similar studies in children [13,14,20], our results were based on a relatively small sample. However, our sample was exclusively composed of children affected by cancer. Note that access to this population and its study presented serious disadvantages compared to other types of populations. Therefore, the findings should be taken with caution as the small size could have affected the results. The prevalence was low, the effects of the treatments were adverse and there was protection from their parents towards them with the aim of isolating them from the prejudices of said disease. Secondly, the feasibility assessed in this study was limited to missing responses. Feasibility should be assessed adding several indicators, particularly in child populations, e.g., completion time, comprehensibility level of the question and participant preferences. Future EQ-5D-Y studies might aim to include such indicators. Third, not all measurement properties were assessed in our study, such as validity, responsiveness and reliability. Future studies are needed to compare these properties in order to suggest users adequately.

Based on our results, this study has implications for clinicians and researchers. The EQ-5D-Y-3L version was previously the most used in the assessment of HRQoL in children, often showing a scarce discriminant power and a high ceiling effect. With the new (until now in beta version) EQ-5D-Y-5L version, it seems that the ceiling effect was reduced, and the questionnaire was better able to detect subjects who selected level one of severity using the 3L version, who subsequently are distributed into level two of severity. Consequently, we recommend the use of the EQ-5D-Y-5L version in future studies and assessments of HRQOL.

## 5. Conclusions

Our findings provided much needed evidence that the EQ-5D-Y-5L version was feasible, having a lower ceiling effect and providing a better distribution of the severity of the problem in the five levels of response compared to those of EQ-5D-Y-3L in the context of chronic illness, which indicated a higher discriminative power. Further research should focus on testing the psychometric properties, comparing among different chronic illness and in the general population, including validity, reliability and responsiveness.

## Figures and Tables

**Table 1:** Patients’ characteristics (n = 72).

Gender	47.9% Males, 52.1% Females	
Age (years)		
6–8	34.2%	
9–11	42.4%	
12–14	23.2%	
Diagnosis	50.7% haematological	
	37.0% solid tumor	
	12.3% brain tumor	
Time since diagnosis (years)		
1	41.1%	
2	32.9%	
3	19.2%	
>3	6.8%	

**Table 2:** Percentages of reported problems in EQ-5D-Y-3L and EQ-5D-Y-5L.

EQ-5D-3L-Y		EQ-5D-Y-5L	
Mobility (Walking About)			
No problems	52 (71.2)	No problems	44 (60.3)
Some problems	20 (27.4)	A little bit of a problem	12 (16.4)
A lot of problems	1 (1.4)	Some problems	11 (15.1)
		A lot of problems	5 (6.8)
		Cannot	1 (1.4)
**Looking after myself**			
No problems	51 (69.9)	No problems	39 (53.4)
Some problems	22 (30.1)	A little bit of a problem	15 (20.5)
A lot of problems	0 (0.0)	Some problems	11 (15.1)
		A lot of problems	8 (11.0)
		Cannot	0 (0.0)
**Doing usual activities**			
No problems	59 (80.8)	No	34 (46.6)
Some problems	13 (17.8)	A little bit of a problem	26 (35.6)
A lot of problems	1 (1.4)	Some problems	9 (12.3)
		A lot of problems	3 (4.1)
		Cannot	1 (1.4)
**Having pain or discomfort**			
No pain or discomfort	57 (78.1)	No pain or discomfort	36 (49.3)
Some pain or discomfort	16 (21.9)	A little bit of pain or discomfort	21 (28.8)
A lot of pain or discomfort	0 (0.0)	Some pain or discomfort	12 (16.4)
		A lot of pain or discomfort	4 (5.5)
		Extreme pain or discomfort	0 (0.0)
**Feeling worried. sad or unhappy**			
Not worried, sad or unhappy	55 (75.3)	Not worried, sad or unhappy	43 (58.9)
A bit worried, sad or unhappy	16 (21.9)	A little bit worried, sad or unhappy	15 (20.5)
Very worried, sad or unhappy	2 (2.7)	Quite worried, sad or unhappy	11 (15.1)
		Really worried, sad or unhappy	4 (5.5)
		Extremely worried, sad or unhappy	0 (0.0)

Note = Data are expressed as frequencies and percentages, n (%).

**Table 3:** Proportions of “no problems” responses for the EQ-5D-3L-Y and EQ-5D-5L-Y systems and ceiling effect change.

Dimension	3L * (n, %)	5L ^†^ (n, %)	Ceiling Effect Reduction	
			Absolute (%)	Relative (%)
Mobility (walking about)	52 (71.2)	44 (60.3)	10.9	15.3
Looking after myself	51 (69.9)	39 (53.4)	16.5	23.6
Doing usual activities	59 (80.8)	34 (46.6)	34.2	42.4
Having pain or discomfort	57 (78.1)	36 (49.3)	28.8	36.9
Feeling worried, sad or unhappy	55 (75.3)	43 (58.9)	16.4	21.8
Overall (11111)	27 (37.0)	15 (20.5)	16.5	44.6

* EQ-5D-Y-3L. ^†^ EQ-5D-Y-5L. Ceiling effect reduction is expressed in absolute (ceiling 3L–ceiling 5L) and relative terms (ceiling 3L–ceiling 5L/ceiling 3L × 100).

**Table 4:** Redistribution properties from EQ-5D-Y-3L to EQ-5D-Y-5L.

D	3L *	5L ^†^	Subgroup	n	Proportions (%)	VAS Mean	VAS Median
**MO**	1	1	g1.1	34	66.6	85.3	90
		2	g1.2	17	33.3	79.1	85
	2	2	g2.2	4	20	76.2	75
		3	g2.3	11	55	60.1	60
		4	g2.4	5	25	59	70
	3	4	g3.4	0	0		
		5	g3.5	1	100	65	65
	inconsistency			1	1.4	90	90
**SC**	1	1	g1.1	39	81.2	85.7	90
		2	g1.2	9	18.7	71.7	80
	2	2	g2.2	6	27.2	59.1	47.5
		3	g2.3	8	36.3	73.7	77.5
		4	g2.4	8	36.3	65.7	70
	3	4	g3.4	0	0.0		
		5	g3.5	0	0.0		
	inconsistency			3	4.1	66.6	65
**UA**	1	1	g1.1	34	58.6	89.8	93.5
		2	g1.2	24	41.3	67.2	70
	2	2	g2.2	2	15.3	60	60
		3	g2.3	8	61.5	69.3	72.5
		4	g2.4	3	23	58.3	70
	3	4	g3.4	0	0.0		
		5	g3.5	1	100	65	65
	inconsistency			1	1.4	80	80
**P/D**	1	1	g1.1	36	64.2	88.2	90
		2	g1.2	20	35.7	67.7	70
	2	2	g2.2	1	6.2	45	45
		3	g2.3	11	68.7	71.8	70
		4	g2.4	4	25	61.2	62.5
	3	4	g3.4	0	0.0		
		5	g3.5	0	0.0		
	inconsistency			1	1.4	52	52
**A/D**	1	1	g1.1	43	81.1	77.6	80
		2	g1.2	10	18.8	80.9	85
	2	2	g2.2	5	31.2	75	80
		3	g2.3	8	50	75.7	75.5
		4	g2.4	3	18.7	84.3	90
	3	4	g3.4	1	100	90	90
		5	g3.5	0	0.0		
	inconsistency			3	4.1	63.3	70

* EQ-5D-Y-3L. ^†^ EQ-5D-Y-5L.

**Table 5:** Absolute and relative informativity of EQ-5D-Y-3L and EQ-5D-Y-5L.

	Absolute Informativity				Relative Informativity	
	3L	5L	Shanon Index *t*-Test		3L	5L
EQ-5D Dimensions	*H*′	*H*′	t	*p*	J′	J′
Mobility (walking about)	4.22	4.13	−0.93	0.34	1.37	1.13
Looking after myself	4.23	4.14	−0.94	0.34	1.37	1.12
Doing usual activities	4.23	4.17	−0.62	0.53	1.42	1.17
Having pain or discomfort	4.23	4.16	−0.71	0.47	1.42	1.16
Feeling worried, sad or unhappy	4.22	4.15	−0.67	0.50	1.37	1.17
Overall system	4.23	4.16	−0.94	0.34	0.94	0.87

## Data Availability

Not applicable.
